# Outcome of COVID-19 in allogeneic stem cell transplant recipients: Results from the EPICOVIDEHA registry

**DOI:** 10.3389/fimmu.2023.1125030

**Published:** 2023-02-24

**Authors:** Alessandro Busca, Jon Salmanton-García, Francesco Marchesi, Francesca Farina, Guldane Cengiz Seval, Jaap Van Doesum, Nick De Jonge, Nathan C. Bahr, Johan Maertens, Joseph Meletiadis, Nicola S. Fracchiolla, Barbora Weinbergerová, Luisa Verga, Zdeněk Ráčil, Moraima Jiménez, Andreas Glenthøj, Ola Blennow, Alina Daniela Tanase, Martin Schönlein, Lucia Prezioso, Nina Khanna, Rafael F. Duarte, Pavel Žák, Marcio Nucci, Marina Machado, Austin Kulasekararaj, Ildefonso Espigado, Elizabeth De Kort, José-María Ribera-Santa Susana, Monia Marchetti, Gabriele Magliano, Iker Falces-Romero, Osman Ilhan, Emanuele Ammatuna, Sofia Zompi, Panagiotis Tsirigotis, Anastasia Antoniadou, Giovanni Paolo Maria Zambrotta, Anna Nordlander, Linda Katharina Karlsson, Michaela Hanakova, Giulia Dragonetti, Alba Cabirta, Caroline Berg Venemyr, Stefanie Gräfe, Jens Van Praet, Athanasios Tragiannidis, Verena Petzer, Alberto López-García, Federico Itri, Ana Groh, Eleni Gavriilaki, Michelina Dargenio, Laman Rahimli, Oliver A. Cornely, Livio Pagano

**Affiliations:** ^1^ Stem Cell Transplant Center, AOU Citta’ della Salute e della Scienza, Turin, Italy; ^2^ University of Cologne, Faculty of Medicine and University Hospital Cologne, Translational Research, Cologne Excellence Cluster on Cellular Stress Responses in Aging-Associated Diseases (CECAD), Cologne, Germany; ^3^ University of Cologne, Faculty of Medicine and University Hospital Cologne, Department I of Internal Medicine, Center for Integrated Oncology Aachen Bonn Cologne Duesseldorf (CIO ABCD) and Excellence Center for Medical Mycology (ECMM), Cologne, Germany; ^4^ Hematology and Stem Cell Transplant Unit, IRCCS Regina Elena National Cancer Institute, Rome, Italy; ^5^ IRCCS Ospedale San Raffaele, Milan, Italy; ^6^ Ankara University, Ankara, Türkiye; ^7^ University Medical Center Groningen, Groningen, Netherlands; ^8^ Amsterdam UMC, Amsterdam, Netherlands; ^9^ University of Kansas Medical Center, Kansas, KS, United States; ^10^ Department of Microbiology, Immunology, and Transplantation, KULeuven, Leuven, Belgium; ^11^ Department of Hematology, UZ Leuven, Leuven, Belgium; ^12^ Clinical Microbiology Laboratory, Medical School, “Attikon” University General Hospital, National and Kapodistrian University of Athens, Athens, Greece; ^13^ Fondazione IRCCS Ca’ Granda Ospedale Maggiore Policlinico, Milan, Italy; ^14^ Department of Internal Medicine - Hematology and Oncology, Masaryk University Hospital Brno, Brno, Czechia; ^15^ Azienda Ospedaliera San Gerardo - Monza, Monza, Italy; ^16^ Università Milano-Bicocca, Milan, Italy; ^17^ Institute of Hematology and Blood Transfusion, Prague, Czechia; ^18^ Department of Hematology, Vall d’Hebron Hospital Universitari, Experimental Hematology, Vall d’Hebron Institute of Oncology (VHIO), Vall d’Hebron Barcelona Hospital, Barcelona, Spain; ^19^ Departament de Medicina, Universitat Autònoma de Barcelona, Barcelona, Spain; ^20^ Department of Hematology, Copenhagen University Hospital - Rigshospitalet, Copenhagen, Denmark; ^21^ Department of Infectious Diseases, Karolinska University Hospital, Stockholm, Sweden; ^22^ Fundeni Clinical Institute, University of Medicine and Pharmacy Carol Davila, Bucharest, Romania; ^23^ Department of Oncology, Hematology and Bone Marrow Transplantation with Section of Pneumology, University Medical Center Hamburg-Eppendorf, Hamburg, Germany; ^24^ Hospital University of Parma - Hematology and Bone Marrow Unit, Parma, Italy; ^25^ Division of Infectious Diseases and Hospital Epidemiology, and Department of Clinical Research, University and University Hospital of Basel, Basel, Switzerland; ^26^ Hospital Universitario Puerta de Hierro, Majadahonda, Spain; ^27^ University Hospital Hradec Králové, Hradec Králové, Czechia; ^28^ Federal University of Rio de Janeiro, Rio de Janeiro, Brazil; ^29^ Clinical Microbiology and Infectious Diseases Department, Hospital General Universitario Gregorio Marañón, Madrid, Spain; ^30^ King’s College Hospital, London, United Kingdom; ^31^ King’s College London, London, United Kingdom; ^32^ Department of Hematology, University Hospital Virgen Macarena - University Hospital Virgen del Rocío, Instituto de Biomedicina de Sevilla (IBIS/CSIC), Universidad de Sevilla (Departamento de Medicina), Seville, Spain; ^33^ Radboudumc, Nijmegen, Netherlands; ^34^ Clinical Hematology Department, ICO-Hospital germans Trias i Pujol, Josep Carreras Research Institute, Badalona, Spain; ^35^ Azienda Ospedaliera Nazionale SS. Antonio e Biagio e Cesare Arrigo, Alessandria, Italy; ^36^ ASST Grande Ospedale Metropolitano Niguarda, Milan, Italy; ^37^ La Paz University Hospital, Madrid, Spain; ^38^ Hematology Unit, Fondazione Policlinico Universitario Agostino Gemelli - IRCCS, Rome, Italy; ^39^ Department of Nephrology and Infectious diseases, AZ Sint-Jan Brugge-Oostende AV, Brugge, Belgium; ^40^ Aristotle University of Thessaloniki, Thessaloniki, Greece; ^41^ Department of Hematology and Oncology, Medical University of Innsbruck, Innsbruck, Austria; ^42^ Fundacion Jimenez Diaz University Hospital, Health Research Institute IIS-FJD, Madrid, Spain; ^43^ San Luigi Gonzaga Hospital - Orbassano, Orbassano, Italy; ^44^ Infektiologie, Universitätsklinikum Frankfurt am Main, Frankfurt am Main, Germany; ^45^ General Hospital of Thessaloniki “George Papanikolaou”, Thessaloniki, Greece; ^46^ Hematology and Stem Cell Transplant Unit, Vito Fazzi, Lecce; ^47^ University of Cologne, Faculty of Medicine and University Hospital Cologne, Clinical Trials Centre Cologne (ZKS Köln), Cologne, Germany; ^48^ University of Cologne, Faculty of Medicine and University Hospital Cologne, Center for Molecular Medicine Cologne (CMMC), Cologne, Germany; ^49^ German Centre for Infection Research (DZIF) , Cologne, Germany; ^50^ Hematology Unit, Università Cattolica del Sacro Cuore, Rome, Italy

**Keywords:** allogeneic HSCT, COVID-19 infection, immunocompromised patients, SARS-CoV-2, hematological malignances

## Abstract

**Background:**

The outcome of COVID-19 in allogeneic hematopoietic stem cell transplantation (HSCT) recipients is almost uniformely considered poor. The aim of present study was to retrospectively analyse the outcome and risk factors for mortality in a large series of patients who developed COVID-19 infection after an allogeneic HSCT.

**Methods:**

This multicenter retrospective study promoted by the European Hematology Association – Infections in Hematology Study Working Group, included 326 adult HSCT patients who had COVID-19 between January 2020 and March 2022.

**Results:**

The median time from HSCT to the diagnosis of COVID-19 was 268 days (IQR 86-713; range 0-185 days). COVID-19 severity was mild in 21% of the patients, severe in 39% and critical in 16% of the patients. In multivariable analysis factors associated with a higher risk of mortality were, age above 50 years, presence of 3 or more comorbidities, active hematologic disease at time of COVID-19 infection, development of COVID-19 within 12 months of HSCT, and severe/critical infections. Overall mortality rate was 21% (n=68): COVID-19 was the main or secondary cause of death in 16% of the patients (n=53).

**Conclusions:**

Mortality in HSCT recipients who develop COVID-19 is high and largely dependent on age, comorbidities, active hematologic disease, timing from transplant and severity of the infection.

## Introduction

Severe acute respiratory syndrome coronavirus 2 (SARS-CoV-2) was recognized in late 2019 and developed into a pandemic with life-threatening disease documented in high-risk groups (1). Allogeneic hematopoietic stem cell transplantation (HSCT) has been increasingly adopted as a curative treatment option for a great variety of hematologic malignancies, however HSCT recipients are vulnerable to viral infections due to neutropenia, immunosuppressive treatments, graft-versus-host disease (GVHD) and incomplete immune reconstitution occurring in the post-transplant period. In this respect, efforts have been made not to postpone transplantation during the pandemic (2). To date, scattered case series of HSCT recipients with coronavirus disease 2019 (COVID-19) have been reported (3–11). Overall, the prognosis of HSCT recipients has been uniformly reported dismal with COVID-19-related mortality ranging between 20 and 39% (5, 6, 12–16), much higher than in the general population. Advanced age, presence of active GVHD and a short time interval from HSCT to COVID-19 were identified as predictors of adverse outcome (3, 5, 14). These findings may be discouraging the treating physicians, fearing for the high fatality rate of HSCT recipients. On the other hand, the use of immunosuppressive agents may potentially mitigate the deleterious systemic inflammatory syndrome secondary to the cytokine storm unleashed by SARS-Cov-2 leading to multiorgan dysfunction and eventually death. According to this observation some studies have reported a lower mortality rate of allogeneic HSCT recipients as compared to non-stem cell transplant patients (18% vs 31%) (3). Hence, aim of our retrospective study was to address the outcome and risk factors for mortality in a large series of patients who developed COVID-19 infection after an allogeneic HSCT.

## Patients and methods

### Study population

This is retrospective multicenter cohort study promoted by the European Hematology Association – Infections in Hematology Study Working Group (EHA-IDWP; EPICOVIDEHA survey, https://pubmed.ncbi.nlm.nih.gov/34235404/). Data have been collected on all consecutive adult patients who received an allogeneic HSCT and had COVID-19 in more than 150 European centers between January 2020 and March 2022. Only patients for whom allogeneic HSCT represented the last treatment performed were included into the study. Each institutional review board independently approved the study.

### Data collection

Researchers at each center collected data using an online questionnaire hosted at www.clinicalsurveys.net, EPICOVIDEHA is registered at http://www.clinicaltrials.gov, with the identifier NCT 04733729. Only de-identified data have been analyzed.

Data collected included: age at transplantation (dichotomized as <50 years and ≥50 years), sex (male *vs* female), time from HSCT to the diagnosis of COVID-19, immunosuppression within 6 months of COVID-19 diagnosis, conditioning intensity (myeloablative *vs* reduced intensity), GVHD prophylaxis, time to engraftment, development of acute or chronic GVHD before COVID-19 diagnosis and immunodeficiency scoring index (ISI) at the time of COVID-19 infection. Clinically significant outcomes (hospital admission and intensive care unit [ICU] admission, vital status) were also evaluated. We did not collect information on treatment strategies of COVID-19.

### Definitions

Confirmed cases of COVID-19 were defined by a positive reverse transcription polymerase chain reaction (RT-PCR) assay of a specimen collected on a nasopharyngeal swab.

The severity of COVID-19 at admission is graded according to the China Center for Disease Control and Prevention definitions (17).

Disease status at the time of SARS-CoV-2 detection was defined according to each specific disease’s revised criteria for leukemia, myeloproliferative neoplasm, multiple myeloma, and lymphoma. The ISI was calculated as previously described (4).

### Endpoints and statistical analysis

The primary outcome of this analysis was overall survival 30 days after COVID-19 diagnosis.

Categorical variables were summarised in frequencies and percentages and continuous variables with median, interquartile range (IQR) and absolute range. Additionally, to determine which factors were associated to mortality in our sample, we performed a Cox regression, with the backwards Wald method. Those variables with a p value <0.1 in the univariable model were included into the multivariable analysis. A p<0.05 was considered significant. Overall survival probability has been plotted in a Kaplan-Meier survival curve. SPSSv25.0 was employed for statistical analyses (SPSS, IBM Corp., Chicago, IL, United States).

## Results

### Demographics

Between January 2020 and March 2022, 326 patients receiving an allogeneic HSCT were diagnosed with COVID-19 infection and registered in EPICOVIDEHA.

Baseline transplant characteristics are shown in **Table 1**. The median age at the time of COVID-19 diagnosis was 51 years (IQR 38-61; range 18-75), 132 patients (41%) were female, and 194 patients (59%) were male. Acute leukemia and myelodysplastic syndromes (MDS) (n=257, 79%) were the most common indications for allogeneic HSCT. Overall, 232 patients (71%) received grafts from alternative donors, including matched unrelated donors in 158 cases (48%) and haploidentical donors in 74 cases (23%). For GVHD prophylaxis, 114 patients (35%) received calcineurin inhibitors-based immunosuppression and 71 patients (22%) received post-transplantation cyclophosphamide (PT/Cy). The majority of the patients (297 out of 326 patients, 91%) were not vaccinated at the time of HSCT.

**Table 1:** Characteristics of HSCT patients with COVID-19 diagnosis.

No. patients	326
Age, median (range), years	51 (18-75)
Sex male/female	194 (60%) / 132 (40%)
Diagnosis	
AML/MDS ALL Lymphomas Multiple myeloma Chronic myeloproliferative malignancies other	196 (60%) 61 (19%) 36 (11%) 5 (2%) 17 (5%) 11 (3%)
Disease status at HSCT	
CR/partial remission stable disease R/R disease unknown	289 (89%) 14 (4%) 15 (5%) 11 (2%)
Conditioning intensity	
myeloablative RIC unknown	224 (69%) 30 (9%) 72 (22%)
Donor	
matched sibling donor MUD Haploidentical unknown	88 (27%) 158 (48%) 74 (23%) 6 (2%)
Graft source BM PBSC CB unknown	28 (9%) 287 (88%) 4 (1%) 7 (2%)
GVHD prophylaxis	
CI plus other PT/Cy other unknown	114 (35%) 71 (22%) 86 (26%) 55 (17%)
Vaccination before HSCT	
no 1 dose 2 doses 3 doses	297 (91%) 3 (1%) 25 (8%) 1 (0.3%
Comorbidities at HSCT*	
1 comorbidity 2 comorbidities 3 comorbidities	95 (29%) 41 (12%) 21 (6%)

HSCT, hematopoietic stem cell transplantation; AML, acute myeloid leukemia; MDS myelodysplastic syndrome; ALL, acute lymphoblastic leukemia; CR, complete remission; R/R relapse/refractory; RIC, reduced intensity conditioning; MUD, matched unrelated donor; BM, bone marrow; PBSC, peripheral blood stem cell; CB, cord blood; CI, calcineurin inhibitors; PT/Cy, post-transplant cyclophosphamide.

*Comorbidities included diabetes, liver disease, renal impairment, smoking history.

### Clinical characteristics

The median time from HSCT to the diagnosis of COVID-19 was 268 days (IQR 86-713; range 0.185 days). Collectively, 45 patients (14%) received post-HSCT vaccination before COVID-19 diagnosis: 6 patients received one dose, 18 patients two doses, 19 patients three doses and 2 patients four doses.

At the time of COVID-19 diagnosis, active grade II-IV acute GVHD (aGVHD) was present in 15 patients (5%), and 32 patients (10%) had evidence of moderate to severe chronic GVHD (cGVHD). Overall, 199 patients (61%) were on systemic immunosuppressive treatments during the last 6 months before COVID-19 diagnosis. Only a minority of patients was neutropenic (ANC ≤500/mm^3^) or lymphocytopenic (ALC ≤200/mm^3^) at the time of COVID-19 diagnosis (6% and 10% respectively).

COVID-19 severity was mild in 21% of the patients, severe in 39% and critical in 16% of the patients; 79 patients (24%) had asymptomatic infection.

Among the 184 patients who were hospitalized, 51 patients (28%) were admitted to ICU, and 34 patients required mechanical ventilation.

Patient characteristics at the time of COVID-19 diagnosis are summarized in **Table 2**.

**Table 2:** Patient Characteristics at diagnosis of COVID-19.

No. patients according to the time of COVID-19 diagnosis	
1^st^ wave (February-June 2020) 2^nd^ wave (September-December 2020) 3^rd^ wave (January-March 2022)	65 (20%) 138 (42%) 123 (38%)
Median time form HSCT to COVID-19, days	268
Disease status at COVID-19 diagnosis	
CR/partial remission	289 (89%)
stable disease	14 (4%)
active disease	18 (5%)
unknown	5 (2%)
Acute GVHD grades II-IV	15 (5%)
Chronic GVHD, moderate to severe	32 (10%)
Patients on systemic immunosuppressive agents	199 (61%)
Symptoms at COVID-19 onset	
Pulmonary	110 (34%)
Pulmonary and extrapulmonary	59 (18%)
Extrapulmonary	74 (23%)
Screening, no symptoms	83 (25%)
Patients requiring hospital admission	184 (56%)
Median duration, days	14
Patients requiring ICU admission	51 (16%)
Median duration, days	15
Immunity subset analysis	
Neutrophils	20 (6%)
≤ 500/mm^3^	20 (6%)
501-999/mm^3^	232 (71%)
≥ 1000/mm^3^	54 (17%)
Unknown	
Lymphocytes	34 (10%)
≤200/mm^3^	43 (13%)
201-499/mm^3^	189 (58%)
≥500/mm^3^	60 (19%)
Unknown	

HSCT, hematopoietic stem cell transplantation; CR, complete remission; GVHD, graft-versus-host disease; ICU, intensive care unit.

### Factors associated with mortality and outcome of COVID-19 infection

The results of Cox regression analysis for factors associated with mortality after COVID-19 diagnosis in allogeneic HSCT recipients are shown in **Table 3**.

**Table 3:** Univariate and multivariate analysis for risk factors associated with COVID-19 mortality.

	Evaluable n	Univariable				Multivariable			
		p value	HR	95% CI		p value	HR	95% CI	
				Lower	Upper			Lower	Upper
Age at HSCT									
<50 years	157	–	–	–	–	–	–	–	
≥50 years	169	**<0.001**	3.880	2.185	6.890	**<0.001**	3.206	1.756	5.853
Time from HSCT to COVID-19									
<12 months ≥12 months	194	–	–	–	–	–	–	–	–
	132	**0.049**	0.596	0.356	0.998	**0.031**	0.538	0.306	0.946
Underlying disease									
Acute leukemia (AML,ALL,MDS) Lymphomas (NHL,HD) Other	257	–	–	–	–				
	36	0.622	0.794	0.317	1.987				
	37	0.317	1.435	0.708	2.910				
Type of HSCT									
Matched sibling donor Alternative (MUD, Haploidentical, Cord) Unknown	88	–	–	–	–				
	232	0.759	0.921	0.547	1.553				
	6	0.961	0.000	0.000	.				
Preparative regimen									
Non-myeloablative RIC Myeloablative Unknown	54	–	–	–	–				
	30	0.819	1.107	0.464	2.644				
	224	0.186	0.663	0.360	1.219				
	18	0.250	1.756	0.673	4.577				
GVHD prophylaxis									
No CI+MTX/MMF PT/Cy Other Unknown	17	–	–	–	–				
	114	0.858	1.116	0.336	3.703				
	71	0.516	0.648	0.175	2.401				
	86	0.519	1.487	0.445	4.974				
	38	0.828	1.156	0.312	4.274				
Comorbidities before COVID-19									
No comorbidities 1-2 comorbidities 3 or more comorbidities	169	–	–	–	–	–	–	–	–
	136	**0.039**	1.720	1.028	2.877	0.164	1.476	0.853	2.553
	21	**0.006**	2.915	1.365	6.225	**0.002**	3.718	1.596	8.662
Disease status at COVID-19 diagnosis									
CR/Partial remission Stable disease Active disease Unknown	289	–	–	–	–	–	–	–	–
	14	0.534	1.382	0.499	3.821	0.475	1.474	0.508	4.275
	18	**<0.001**	4.255	2.092	8.652	**<0.001**	3.859	1.810	8.226
	5	0.056	3.968	0.963	16.353	0.282	2.239	0.516	9.713
ISI group									
Low risk Moderate/High risk	149	–	–	–	–				
	177	0.250	1.332	0.817	2.173				
Acute GVHD before COVID-19 diagnosis									
0-I II-IV Unknown	237	–	–	–	–				
	74	0.766	1.092	0.611	1.951				
	15	0.203	1.820	0.724	4.576				
Chronic GVHD before COVID-19 diagnosis									
Absent-Mild Moderate-Severe Unknown	261	–	–	–	–				
	48	0.797	1.089	0.570	2.080				
	17	0.141	0.227	0.031	1.639				
COVID-19 severity									
Asymptomatic Mild infection Severe infection Critical infection	79	–	–	–	–	–	–	–	–
	68	0.253	1.953	0.620	6.156	0.192	2.160	0.680	6.857
	128	**0.014**	3.363	1.273	8.887	**0.012**	3.628	1.333	9.879
	51	**<0.001**	15.27	5.954	39.157	**<0.001**	12.91	4.892	34.069
Drugs within 6 months of COVID-19 diagnosis									
None Immunosuppressive/Corticosteroids Unknown	66	–	–	–	–				
	221	0.198	1.596	0.784	3.250				
	39	0.226	1.744	0.708	4.292				

HSCT, hematopoietic stem cell transplantation; AML, acute myeloid leukemia; MDS myelodysplastic syndrome; ALL, acute lymphoblastic leukemia; NHL, Non-Hodgkin lymphoma; HD, Hodgkin lymphoma; MUD, matched unrelated donor; RIC, reduced intensity conditioning; GVHD, graft-versus-host disease; CI, calcineurin inhibitors; MTX, methotrexate; MMF, mycophenolate mofetil; PT/Cy, post-transplant cyclophosphamide; CR, complete remission; ISI, immunodeficiency scoring index.

In multivariable analysis factors associated with a higher risk of mortality were, age 50 years or older, presence of 3 or more comorbidities, active hematologic disease at time of COVID-19 infection, development of COVID-19 within 12 months of HSCT, and severe/critical infections. The type of HSCT, the intensity of preparative regimen, the presence of aGVHD and cGVHD before COVID-19 infection and ISI group were not associated with increased mortality.

Vaccination and the number of doses (one, two or more doses) administered did not have an impact on the outcome of the patients.

**Figure 1:**
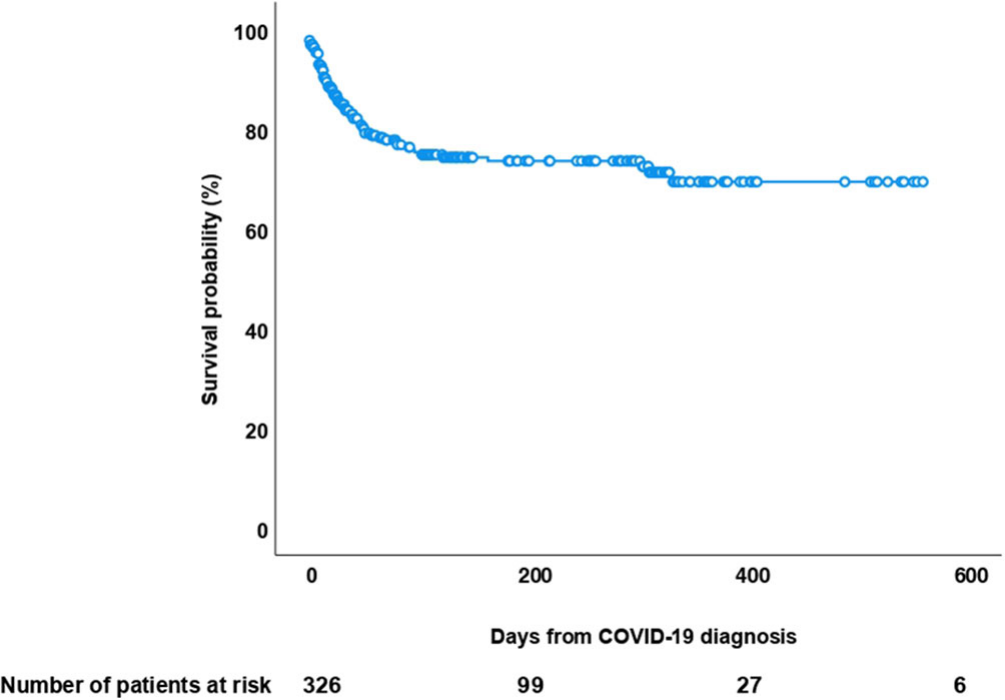
Kaplan-Meier overall survival curve after diagnosis of COVID-19 infection in 326 allogeneic HSCT recipients.

At the time of last follow-up, 258 patients (79%) are alive: from the diagnosis of COVID-19, the median follow of survivors was 126 days (IQR 32-339; range 0-643 days). The Kaplan-Meier overall survival estimate is shown in **Figure 1**. Overall, 68 patients (21%) had died after a median follow-up post COVID-19 of 26 days (IQR 12-56; range 0-379 days). Causes of death were COVID-19 infection in 42 cases (13%), COVID-19 in parallel to recurrence of the underlying hematologic malignancy in 11 cases (3%), and hematologic malignancy +/- other reasons in 15 cases (5%).

## Discussion

To our knowledge, the present study includes the largest series of allogeneic HSCT recipients with COVID-19. The aim of our analysis was to evaluate the outcome of COVID-19 in a cohort of patients particularly susceptible to infectious complications and to investigate risk factors that may impact on mortality. Patients were at high risk for severe disease and adverse outcome from COVID-19: a large proportion of patients (70%) received HSCT from alternative donors, 16% had critical infection and required ICU admission. Even more importantly, the median time from HSCT to COVID-19 infection was 268 days, remarkably shorter when compared to other reports (474-790 days) (3, 4, 12, 14). This finding might mirror the adoption of less stringent measures of stewardship among transplant centers after the 1^st^ and 2^nd^ COVID-19 wave. In fact, it should be recognized that one third of the patients had COVID-19 infection diagnosed during the 3^rd^ wave.

Our study demonstrated that COVID-19 infection is a severe complication in allogeneic HSCT recipients with an attributable mortality of 16%. Our results are roughly superimposable to those reported from other recent studies. The CIBMTR reported a COVID-19-related mortality rate of 20% in 184 allogeneic HSCT recipients (14), Piñana et al. analyzed the outcome of COVID-19 in 65 allogeneic HSCT patients and reported a mortality of 18% (3). An EBMT (European Society for Blood and Marrow Transplantation) and GETH (Spanish Group of Hematopoietic Stem Cell Transplantation) prospective study reported an overall mortality rate of 28% among 236 allogeneic HSCT recipients (12). Other smaller series documented similar mortality rates ranging from 20 to 25% (5–7, 9). It is worthwhile recalling that mortality in the general population declined over the 2^nd^ and 3^rd^ waves: this observation should be considered when we consider that only 20% of our patients were diagnosed with COVID-19 during the first wave.

Multivariable analysis showed that age, comorbidities, active disease, and severe/critical infection have been associated with higher mortality, consistent with previous studies (3, 4, 12, 14). Nevertheless, it should be noted that a consistent number of comorbidities and demographic characteristics (i.e. diabetes, BMI, race) potentially influencing the outcome of the patients were not available Similarly, time from HSCT to COVID-19 diagnosis of less than 12 months was a factor significantly associated with fatal outcome. Surprisingly, GVHD at the time of COVID-19 diagnosis did not impact on the outcome of our patients, however it should be underscored that only 5% of the patients had grade II-IV acute GVHD and 10% had moderate/severe chronic GVHD; in addition, data on the cumulative dose of steroids and the different immunosuppressive agents used were not available.

Likewise, mortality was not influenced by the use of immunosuppressive drugs and steroids during the 6 months preceding COVID-19 diagnosis, notwithstanding the number of patients on treatment was extremely high. Whether the administration of two to three doses of vaccine in 20% of the patients might have contributed to dampen the severity of the disease as documented in some studies (18, 19), remains speculative. Regrettably, we do not have data on patient seroconversion and B-cell reconstitution underpinning the effectiveness of vaccination.

ISI did not result as an independent risk factor for poor outcome as shown by Ljungman et al, however several variables included in the ISI (ANC and ALC, GVHD) were poorly represented in our study group. Two studies including a small number of patients reported a favorable outcome of patients receiving PT/Cy as GVHD prophylaxis, since cytokine release syndrome (CRS) associated with haploidentical HSCT and COVID-19 share similar pathophysiology (10, 11). It is well known that a dysregulated, excessive immune response with increased pro-inflammatory cytokines levels during the later phases of COVID-19 infection may lead to multiorgan failure (20). Cy is an alkylating agent largely used in the setting of haploidentical HSCT due to its capacity of depleting rapidly proliferating T-cells while sparing regulatory-T cells (21). According to these observations, it has been postulated a potential effect of Cy in mitigating the COVID-19 infection (22), however in our cohort, we did not find any significant relationship between the type of GVHD prophylaxis and mortality, including PT/Cy in 22% of the cases. ANC and ALC at the time of COVID-19 diagnosis did not result as significant factors for disease severity as shown in other studies (3, 5), however a minority of our patients were neutropenic or had low ALC.

We recognize limitations of our study inherent to the retrospective design. We cannot exclude a possible selection bias in the registration of the patients among such a large number of centers. Our study presents a significant heterogeneity in terms of different variants of concern, namely wild type, delta and omicron variants, each with distinctive transmission rates and infection associated mortality. Information on the treatment of COVID-19 were not available and the management of patients with COVID-19 across centers may differ profoundly, potentially influencing clinical outcome.

## Conclusion

Our study on a large number of patients who developed COVID-19 infection following HSCT, shows a high mortality rate compared to the general population. In this respect, it is of upmost relevance to see whether vaccination of patients after HSCT and the availability of pre- and post-exposure prophylactic measures effectively mitigates the severity of the disease in this vulnerable group of patients.

## Data availability statement

The raw data supporting the conclusions of this article will be made available by the authors, without undue reservation.

## Ethics statement

The studies involving human participants were reviewed and approved by EPICOVIDEHA was approved by the local ethics committee of the Fondazione Policlinico Universitario Agostino Gemelli - IRCCS, Università Cattolica del Sacro Cuore of Rome, Italy (Study ID: 3226). The patients/participants provided their written informed consent to participate in this study.

## A list of EPICOVIDEHA Collaborators members

Juergen Prattes, Malgorzata Mikulska, Gustavo-Adolfo Méndez, Tobias Lahmer, Pavel Jindra, Anna Guidetti, Rita Fazzi, Maria Ilaria Del Principe, Cristina De Ramón, Maria Calbacho, Zlate Stojanoski, Andrés Soto, Alexandra Serris, Irati Ormazabal-Vélez, Ali S. Omrani, Milan Navrátil, Sonia Martín-Pérez, Joyce Marques De Almeida, Sylvain Lamure, Martin Kolditz, Ozren Jaksic, Martin Hoenigl, Carolina Garcia-Vidal, Noemí Fernández, Shaimaa El-Ashwah, Natasha Čolović, Martin Čerňan, Caterina Buquicchio, Valentina Bonuomo, Josip Batinić, Murtadha Al-Khabori, Tatjana Adžić-Vukičević, Juan-Alberto Martín-González, Maria Vittoria Sacchi, María-Josefa Jiménez-Lorenzo, Dominik Wolf, Maria Vehreschild, Raul Cordoba, Ramón García-Sanz, Toni Valković, Miloš Mladenović, Nicole García-Poutón, Ziad Emarah, Julio Dávila-Valls

## Author contributions

AB, JS-G, FM, OC and LiP contributed to study design, study supervision, and data interpretation and wrote the paper. AB, JS-G and LiP did the statistical plan. JS-G performed the analysis and AB, JS-G and LiP interpreted the data. All authors recruited participants and collected and interpreted data, contributed to manuscript writing and review of the manuscript, agreed to be accountable for all aspects of the work in ensuring that questions related to the accuracy or integrity of any part of the work are appropriately investigated and resolved and have read and agreed to the published version of the manuscript. All authors have read and agreed to the published version of the manuscript. 
